# Reassessing lidocaine as an electroporation sensitizer in vitro

**DOI:** 10.1038/s41598-025-11695-3

**Published:** 2025-07-15

**Authors:** Anja Blažič, Rok Šmerc, Tamara Polajžer, Damijan Miklavčič, Lea Rems

**Affiliations:** Faculty of Electrical Engineering, University of Ljubljana, Ljubljana, 1000 Slovenia

**Keywords:** Electroporation, Sensitization, Electropermeabilization, Cell survival, Lidocaine, Melanoma cells, Cell biology, Medical research

## Abstract

High-intensity pulsed electric fields induce transient increase in membrane permeability, a phenomenon known as electroporation, with broad applications in medicine, including electrochemotherapy (ECT), gene electrotransfer and tissue ablation. As electroporation technologies become increasingly established in clinical practice, understanding how commonly used pharmacological agents influence treatment outcomes is gaining importance. Lidocaine, a widely used local anesthetic and ion channel modulator, has recently been investigated as a potential sensitizer to enhance the efficacy of electroporation. Here, we examined the effects of lidocaine on membrane permeabilization and cell viability using standard 8 × 100 µs ECT pulses across four cell lines: melanoma B16-F1, myoblast C2C12, CHO-K1 cells with low ion channel expression, and NS-HEK cells with stable Na_v_1.5 expression. We show that 10 mM lidocaine has only modest effects on electroporation outcomes, while 35 mM considerably lowers the electric field threshold for irreversible electroporation by 25–40% in melanoma cells. However, concentrations of even 10 mM exceed those reported in tissues following local administration of lidocaine. This questions the clinical relevance of lidocaine’s sensitization effect and warrants further investigation. Our study also highlights the importance of evaluating drug–electroporation interactions under rigorously controlled experimental conditions to ensure meaningful translation into clinical applications.

**Supplementary Information:**

The online version contains supplementary material available at 10.1038/s41598-025-11695-3.

## Introduction

High-intensity pulsed electric fields are widely used in medicine^1^ as well as in biotechnology^2^ and food technology^3^ to achieve a transient increase in cell membrane permeability, a phenomenon known as electroporation. Among the most developed clinical applications are electrochemotherapy, irreversible electroporation for tissue ablation (tumor, cardiac), and gene electrotransfer^4–6^. The extent of electroporation depends primarily on the pulse parameters—including duration, amplitude, and repetition rate—which determine whether the process is reversible, allowing cell survival, or irreversible, leading to cell death^7^. Sensitization methods can be used to increase the extent of membrane permeabilization or cell death under fixed pulse parameters. These methods include the addition of surfactants and the application of hypotonic shock^8,9^. Additionally, they can involve modifications to pulse delivery, such as dividing a train of pulses into two shorter sequences administered several minutes apart, although this approach is not applicable to all conditions^10,11^.

Pharmacological agents like lidocaine have also been explored as sensitizers^12–14^. Lidocaine is an ion channel modulator that primarily inhibits voltage-gated sodium channels but also interacts with other membrane proteins and influences the biophysical properties of the membrane^15,16^. It is used as a local anesthetic and, at lower concentrations, as class 1b antiarrhythmic drug^17^. Recent studies have demonstrated that lidocaine can considerably modulate electroporation outcomes. Specifically, Grys et al.^12^ showed that 10 mM lidocaine reduced the electric field thresholds for reversible and irreversible electroporation in cancer cells and human fibroblasts. Similarly, Sherba et al.^13^ observed that 10 mM lidocaine enhanced irreversible electroporation in mouse fibroblasts. An in vivo study by Pan et al.^14^ further indicated that intra-arterial (directly into the hepatic artery) administration of 5 mg/ml (18.5 mM, 0.5%) before and 5 mg/ml during pulse application could expand irreversibly electroporated zones in porcine liver.

The intriguing effect of lidocaine in lowering electroporation thresholds (i.e., increasing the electroporation zone) has potentially important implications for electrochemotherapy (ECT). According to standard operating procedures for ECT, lidocaine is used as a local anesthetic to provide pain relief during the treatment of cutaneous tumors and skin metastases^18,19^. Similarly, lidocaine is used as anesthetic before gene electrotransfer (GET) for gene therapy applications^20,21^. However, previous studies employed experimental conditions that were not directly relevant to typical ECT and GET conditions. The in vitro experiments used a low conductivity electroporation solution (< 1 mS/cm); while such solution may approximate the conductivity of certain low-conductive tissues (e.g. bone and fat), it does not represent the ionic environment relevant to tumor or muscle tissues, which are typical targets of ECT and GET, respectively^22^. The pulse parameters also differed from the clinical ECT protocol (8 × 100 µs pulses applied at a repetition rate of 1 Hz)^18,19^. In vitro studies by Grys et al.^12^ and Sherba et al.^13^ used single pulses with duration of 80‒620 ms and 0.06‒1.00 ms, respectively. The in vivo study by Pan et al.^14^ used conventional irreversible electroporation pulse protocol applying 90 pulses of 90 µs. Additionally, as lidocaine is primarily metabolized in the liver^23^, this could potentially influence the in vivo results.

Building on previous studies, we investigated how the presence of lidocaine affects membrane permeability and cell survival when cells are exposed to conventional ECT pulses in vitro. We selected four different cell lines: B16-F1 mouse melanoma cells to reflect the clinical application of ECT in cutaneous tumors and skin metastases^18,19^; C2C12 mouse myoblasts as a model for GET^24^; CHO-K1 cells as a model of cells with low endogenous ion channel expression^25^; and NS-HEK cells with stable expression of Na_V_1.5 channels^26,27^ to explore the potential role of sodium channels in the observed outcomes. We performed experiments using both a low conductivity solution and a Tyrode solution that mimics the typical conductivity and ionic composition of extracellular fluids^28^. Using a 10 min lidocaine incubation period^12^, we tested two concentrations: 10 mM (0.3%), aligned with previous in vitro research^12,13^, and 35 mM (1%), corresponding to standard anesthetic injection dosage. Overall, our findings indicate that lidocaine has modest effects on membrane permeabilization and cell survival at 10 mM, while a pronounced reduction in survival was observed only at a higher concentration of 35 mM.

## Results

**Fig. 1:**
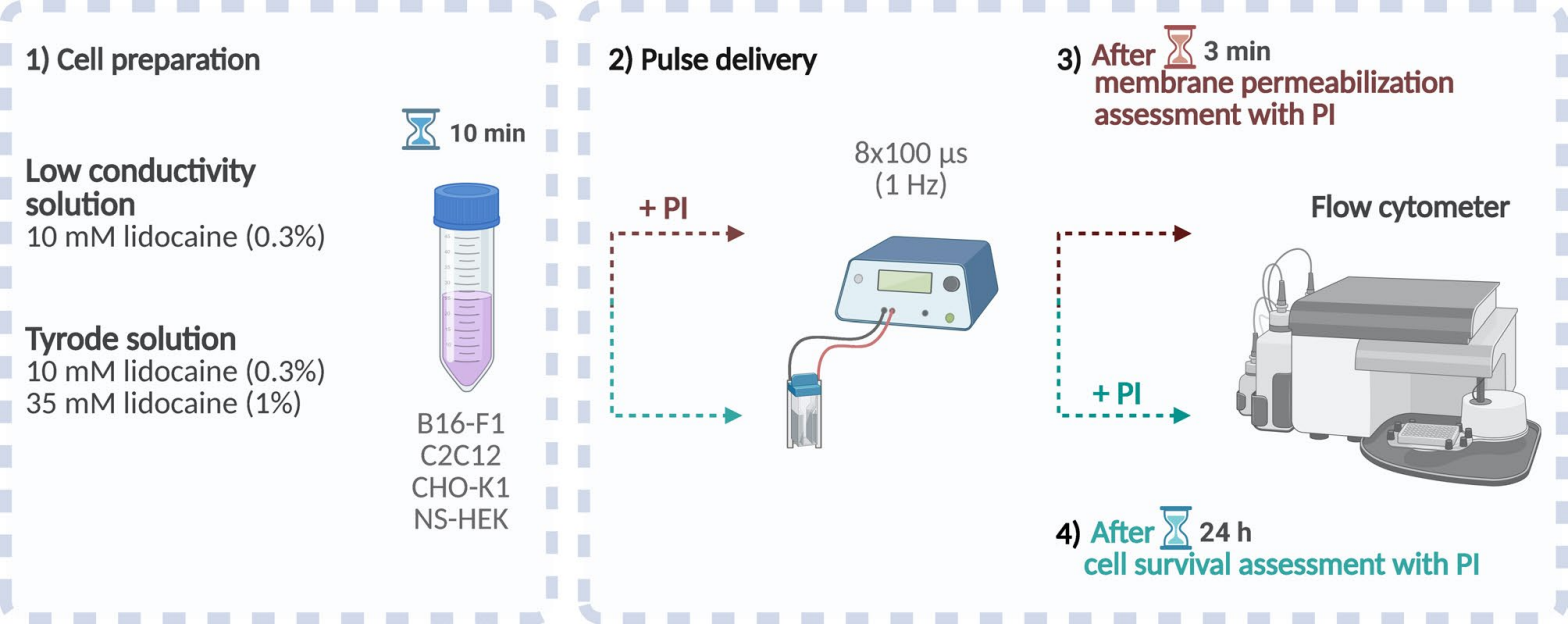
Schematic representation of the experimental design. The workflow consisted of four parts: (1) Incubation of cells (B16-F1, C2C12, CHO-K1, or NS-HEK) with or without lidocaine in the chosen electroporation solution for 10 min; (2) Application of eight 100 µs electric pulses at 1 Hz, in the presence of propidium iodide (PI) for membrane permeabilization analysis or in its absence for cell survival analysis; (3) Measurement of membrane permeabilization by PI uptake 3 min after electroporation using flow cytometry; and (4) Assessment of cell survival 24 h after electroporation by staining with PI and counting unstained live cells with flow cytometry. Permeabilization and survival were assessed on separate samples. Created with BioRender.com.

Our experiments were designed to evaluate the effect of lidocaine (in hydrocholoride salt form) on membrane permeabilization and cell survival following electroporation. The experimental protocol consisted of four parts, as shown in Fig. 1: (1) B16-F1, C2C12, CHO-K1, or NS-HEK cells were first suspended in the chosen electroporation solution with or without lidocaine and incubated for 10 min. (2) Cells were then exposed to 8 × 100 µs pulses of chosen amplitude delivered at 1 Hz in standard electroporation cuvettes, either in the presence of propidium iodide (PI; for permeabilization analysis) or its absence (for cell survival assessment); (3) Membrane permeabilization was assessed based on PI uptake using flow cytometer, 3 min after electroporation. PI is a nucleic acid stain that selectively enters cells with compromised membranes. It is typically used for assessing cell survival but is commonly applied in electroporation experiments to evaluate membrane permeabilization^29,30^; (4) For cell survival assessment, cells were centrifuged following pulse delivery, resuspended in growth medium, plated and incubated for 24 h. Afterwards, dead cells were stained with PI, and non-stained live cells were counted using flow cytometry to determine cell survival. Note that permeabilization and survival were assessed on separate samples.

### Effect of 10 mM lidocaine on membrane permeabilization in low conductivity electroporation solution

**Fig. 2:**
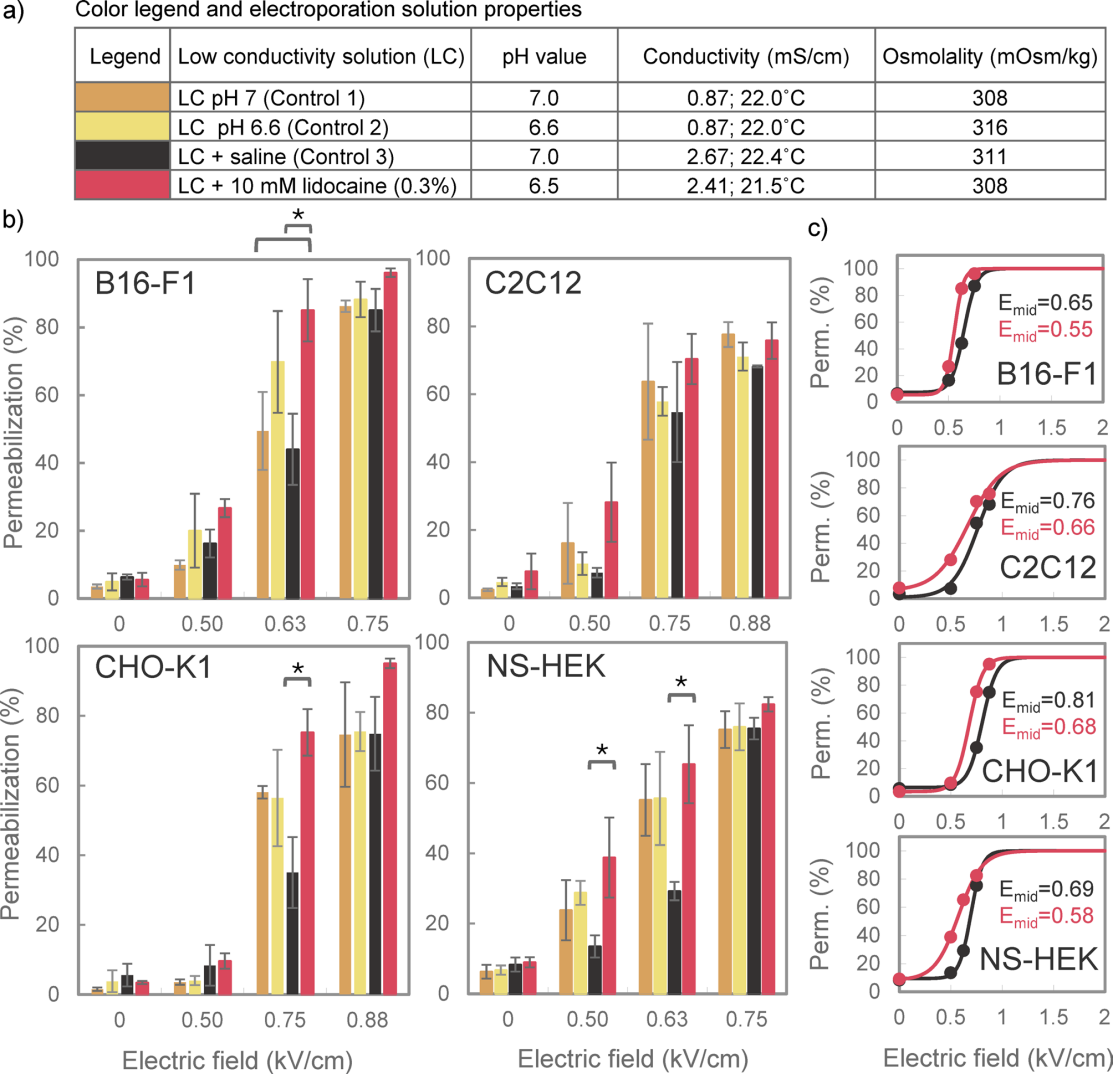
The effect of 10 mM lidocaine on membrane permeabilization in low conductivity (LC) electroporation solution. (a) Table listing all tested electroporation solutions with their pH, conductivity and osmolality values, and the corresponding color legend for panels b and c. (b) The percentage of permeabilized cells was assessed using four different cell lines (B16-F1, C2C12, CHO-K1 and NS-HEK) after exposure to 8 × 100 µs pulses (1 Hz). Results are presented as mean ± SD (*N* = 3), with statistically significant differences indicated by * (*p* < 0.05, One-way ANOVA or ANOVA on ranks). Note that the electric field strength on the x-axis does not scale linearly. (c) Sigmoidal curves, fitted to data for LC + saline (black line) and LC + 10 mM lidocaine (red line) from panel b, showing the relationship between electric field strength and membrane permeabilization. Dots indicate the mean values from panel b. Fits were obtained using the least-square method in Matlab 2021b (MathWorks, USA). *E*_*mid*_ represents the electric field strength (in kV/cm) at the inflection point, where approximately 50% of the cells became permeabilized.

We first evaluated how 10 mM lidocaine (0.3%) affects membrane permeabilization in a low conductivity (LC) solution, which was used in a previous in vitro study investigating the effect of lidocaine on the electroporation outcome^12^. We observed that adding 10 mM lidocaine to this LC solution caused two changes: a decrease in pH from 7.0 to 6.6 and an increase in conductivity from 0.87 mS/cm to 2.41 mS/cm, while the osmolality remained practically unchanged. To better understand the impact of pH reduction and conductivity increase, we prepared two additional LC solutions: one in which pH was decreased using 1 M HCl solution, and another in which conductivity was increased with physiological saline (0.9% NaCl), hereafter referred to as saline. All media’s pH, conductivity, and osmolality values are presented in Fig. 2a. For each of the four tested cell lines (B16-F1, C2C12, CHO-K1, and NS-HEK) we thus had three control and one experimental group based on the electroporation solution: LC pH 7 (Control 1), LC pH 6.5 (Control 2), LC + saline (Control 3), and LC + 10 mM lidocaine.

Results presented in Fig. 2b indicate that pH variations had no effect on membrane permeabilization, as no significant differences were observed between LC pH 7 (Control 1) and LC pH 6.5 (Control 2) in none of the cell lines. Similarly, no significant differences were found between LC pH 7 (Control 1) and LC + saline (Control 3), though we observed somewhat reduced permeabilization in solution with higher conductivity (Control 3) under some conditions (in CHO-K1 cells at 0.75 kV/cm, and in NS-HEK cells at 0.50 and 0.63 kV/cm). This aligns with previous studies showing that electroporation is enhanced in solutions with lower conductivity^31,32^. Adding 10 mM lidocaine significantly increased membrane permeabilization compared to LC pH 7 (Control 1), but only in B16-F1 cells at 0.63 kV/cm. Nevertheless, lidocaine-mediated increase in permeabilization became more evident when compared to the control with similar conductivity, i.e., LC + saline (Control 3). This increase was observed across all cell lines and reached statistical significance in B16-F1 at 0.63 kV/cm, CHO-K1 at 0.75 kV/cm, and NS-HEK at 0.50 kV/cm and 0.63 kV/cm. At these specific electric field strengths, lidocaine increased the percentage of permeabilized cells by up to ~ 40% compared to control.

The greatest differences in permeabilization between lidocaine-treated and control groups were observed at intermediate electric fields, where the percentage of permeabilized cells was rapidly increasing with electric field strength. To better illustrate how lidocaine affected the functional relationship between the electric field strength and the percentage of permeabilized cells, we fitted data from LC + 10 mM lidocaine and LC + saline to sigmoidal curves, presented in Fig. 2c. Across all cell lines, lidocaine shifted the curves towards lower electric field strengths, but only to a modest extent. The electric field strength at the inflection point (*E*_*mid*_, where approximately half of the cells became permeabilized) decreased by 15%, 13%, 16%, and 16% in B16-F1, C2C12, CHO-K1, and NS-HEK, respectively.

These initial experiments demonstrated the importance of designing an appropriate control for assessing drug-specific effects. Saline is commonly used as a placebo or solvent in clinical and in vivo studies^14,33^. It has similar conductivity as the stock lidocaine hydrochloride solution (69.3 mM, 2%) that was added to the samples in our experiments and its main anion is chloride (same as in lidocaine hydrochloride solution). Furthermore, addition of equal volume of saline or lidocaine solution dilutes the other components of the electroporation solution in the same proportion. Thus, we performed all subsequent experiments using saline as “placebo” to assess the effect of lidocaine on electroporation outcome.

### Effect of 10 mM lidocaine on membrane permeabilization in Tyrode solution

**Fig. 3:**
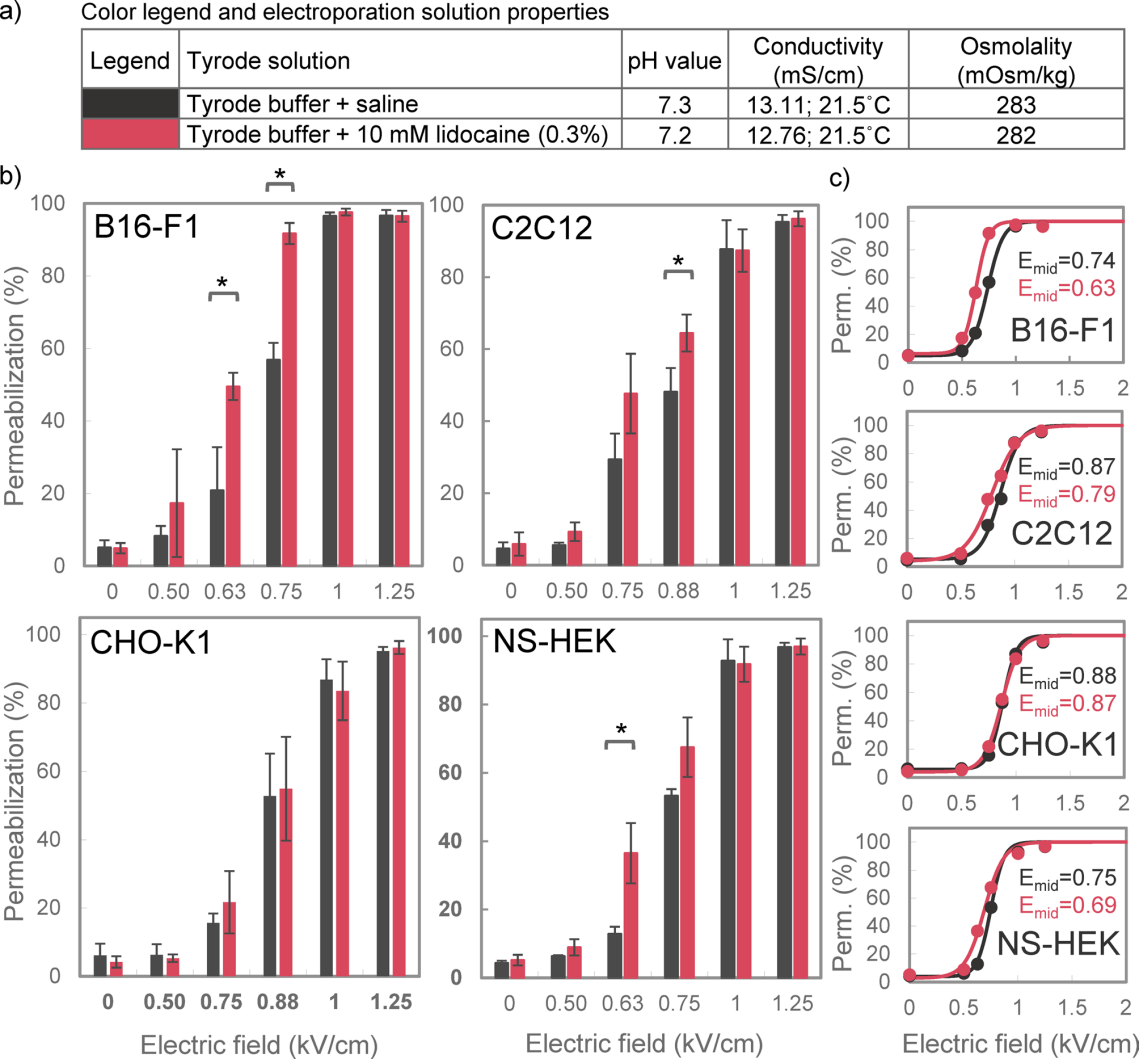
The effect of 10 mM lidocaine on membrane permeabilization in Tyrode solution. (a) Table listing all tested electroporation solutions with their pH, conductivity and osmolality values, and the corresponding color legend for panels b and c. (b) The percentage of permeabilized cells was assessed using four different cell lines (B16-F1, C2C12, CHO-K1 and NS-HEK) after exposure to 8 × 100 µs pulses (1 Hz). Results are presented as mean ± SD (*N* = 3), with statistically significant differences indicated by * (*p* < 0.05, One-way ANOVA or ANOVA on ranks). Note that the electric field strength on the x-axis does not scale linearly. (c) Sigmoidal curves, fitted to the data in panel b, showing the relationship between electric field strength and membrane permeabilization. Dots indicate the mean values from panel b. *E*_*mid*_ represents the electric field strength (in kV/cm) at the inflection point.

Further experiments were conducted in a Tyrode solution that has approximately the same conductivity as saline and is ~ 15× more conductive than the LC solution. We compared two groups: Tyrode solution with saline and Tyrode solution with 10 mM lidocaine. In both groups the electroporation solution had very similar pH, conductivity, and osmolality values (Fig. 3a). 10 mM lidocaine significantly increased cell membrane permeabilization compared to control in B16-F1 at 0.63 kV/cm and 0.75 kV/cm, in C2C12 at 0.88 kV/cm, and in NS-HEK cells at 0.63 kV/cm, but not in CHO-K1 cells (Fig. 3b). Sigmoidal curves fitted to the data are presented in Fig. 3c. The electric field strength at the inflection point (*E*_*mid*_) decreased in the presence of lidocaine by 15%, 9%, 8%, and 1% in B16-F1, C2C12, NS-HEK, and CHO-K1, respectively.

### Effect of 10 mM lidocaine on cell survival in Tyrode solution

**Fig. 4:**
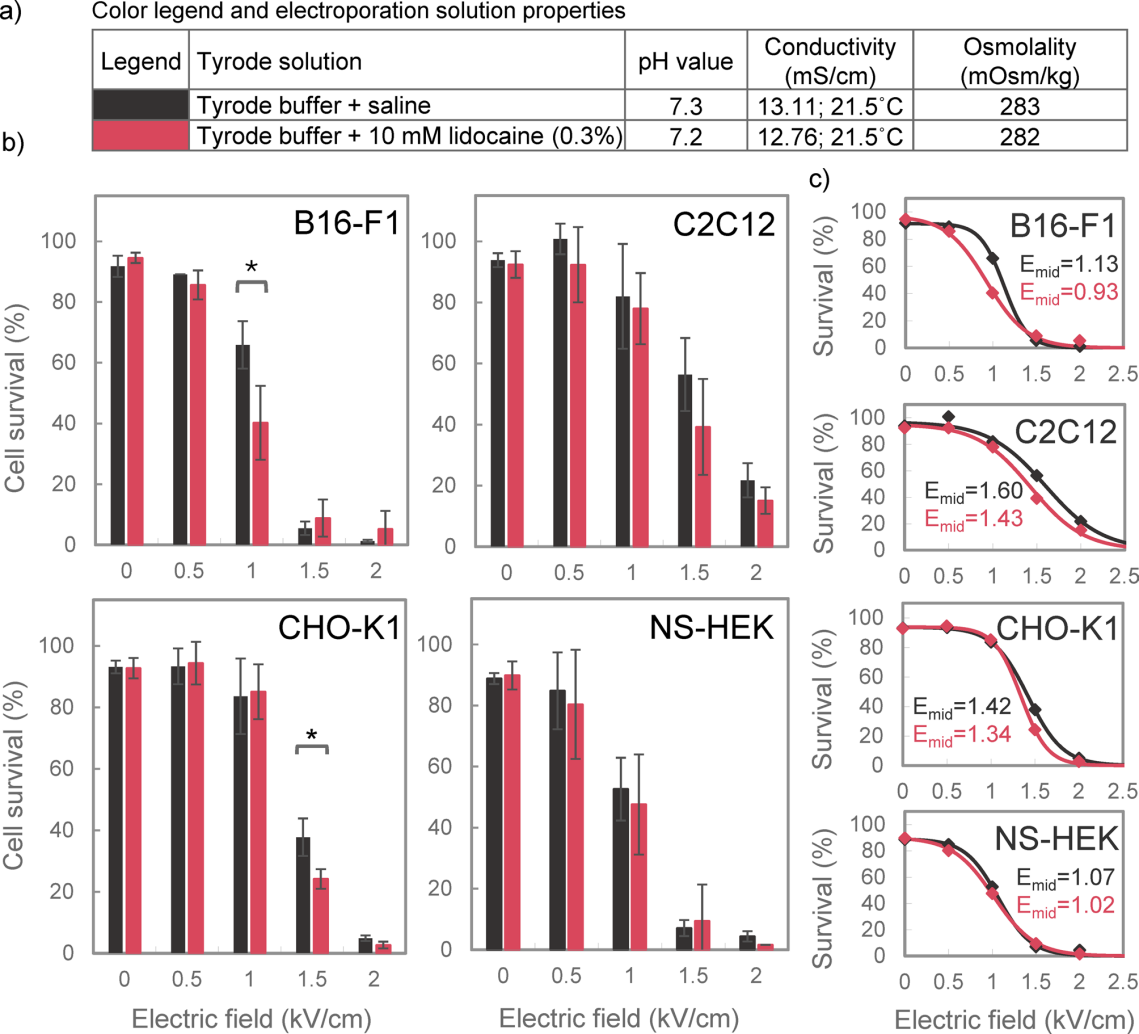
The effect of 10 mM lidocaine on cell survival in Tyrode solution. (a) Table listing all tested electroporation solutions with their pH, conductivity and osmolality values, and the corresponding color legend for panels b and c. (b) The percentage of survived cells was assessed using four different cell lines (B16-F1, C2C12, CHO-K1 and NS-HEK) after exposure to 8 × 100 µs pulses (1 Hz). Results are presented as mean ± SD (*N* = 3), with statistically significant differences indicated by * (*p* < 0.05, One-way ANOVA or ANOVA on ranks). (c) Sigmoidal curves, fitted to the data in panel b, showing the relationship between electric field strength and cell survival. Dots indicate the mean values from panel b. *E*_*mid*_ represents the electric field strength (in kV/cm) at the inflection point.

The effect of 10 mM lidocaine on cell survival was assessed using PI, as explained in Fig. 1. We used a wider range of pulse amplitudes, compared to permeabilization experiments, to achieve cell survival close to 0%. Our results (Fig. 4b) demonstrated that 10 mM lidocaine significantly reduced survival in B16-F1 cells at 1 kV/cm and CHO-K1 cells at 1.5 kV/cm, but not in C2C12 and NS-HEK cells. Fits to sigmoidal curves (Fig. 4c) showed that lidocaine decreased the *E*_*mid*_ for survival by 18%, 11%, 6%, and 5% in B16-F1, C2C12, CHO-K1, and NS-HEK, respectively.

### Effect of 35 mM lidocaine on permeabilization and survival in melanoma cells

**Fig. 5:**
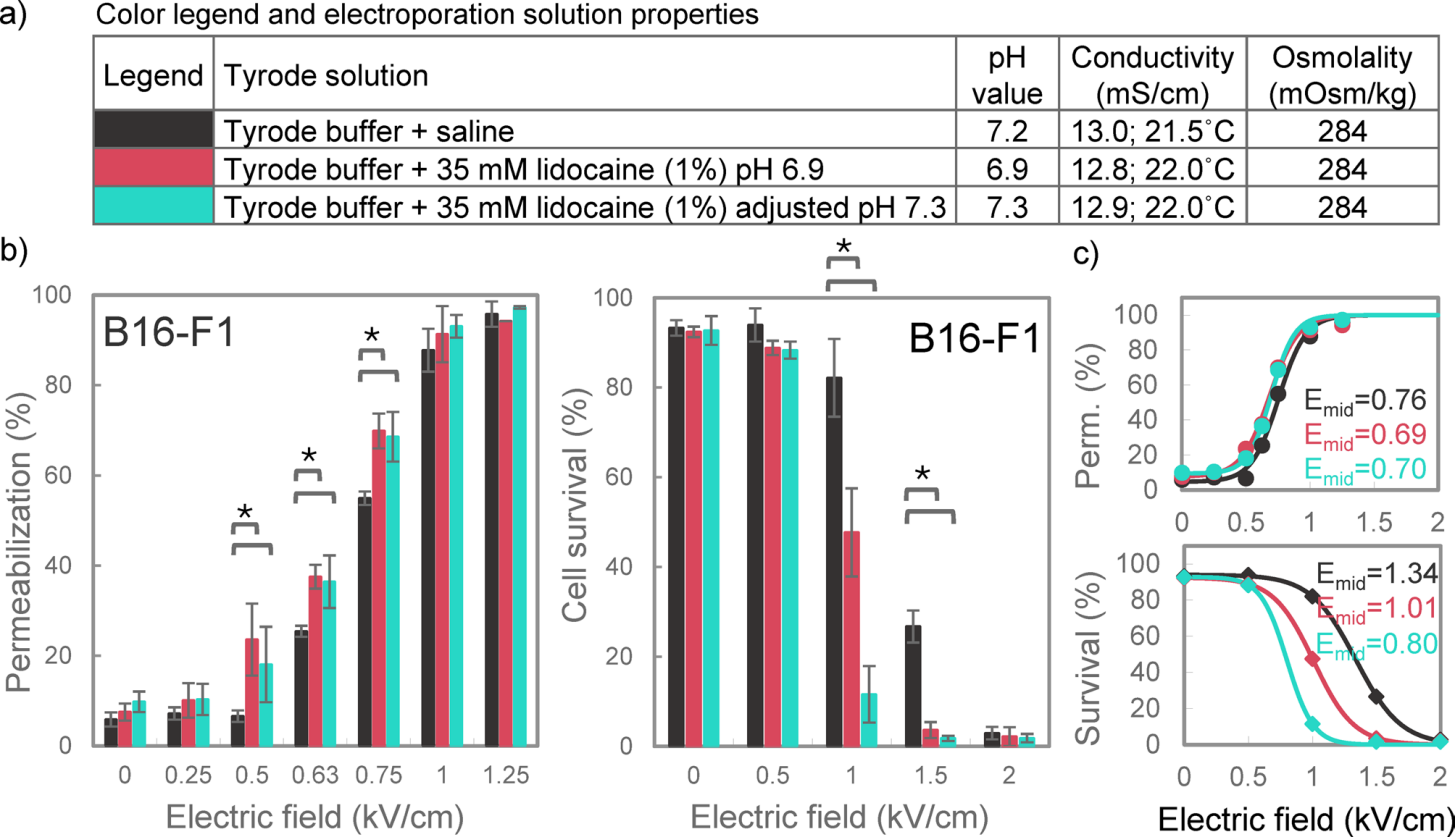
The effect of 35 mM lidocaine on membrane permeabilization and cell survival in Tyrode solution. (a) Table listing all tested electroporation solutions with their pH, conductivity and osmolality values, and the corresponding color legend for panels b and c. (b) Percentage of permeabilized and percentage of live B16-F1 cells assessed after pulse exposure to 8 × 100 µs pulses in the presence of 35 mM (1%) lidocaine. Results are presented as mean ± SD (*N* = 3), with statistically significant differences indicated by * (*p* < 0.05, One-way ANOVA). (c) Sigmoidal curves, fitted to data in panel b, showing the relationship between electric field strength and membrane permeabilization or cell survival. Dots indicate the mean values from panel b. *E*_*mid*_ represents the electric field strength (in kV/cm) at the inflection point.

B16-F1 cells were selected for further investigation because they showed the most pronounced sensitization effect with 10 mM lidocaine. To assess whether this effect could be enhanced, we tested a higher lidocaine concentration of 35 mM (1%). While the addition of 35 mM lidocaine did not affect the conductivity or osmolality of the Tyrode solution, it lowered its pH to 6.9 (Fig. 5a). To account for this pH change, we added another experimental group with 35 mM lidocaine, where the pH was adjusted to 7.3 using NaOH. As shown in Fig. 5b, the presence of 35 mM lidocaine significantly increased membrane permeabilization in both 35 mM lidocaine groups, regardless of pH adjustment, at 0.5 kV/cm, 0.63 kV/cm, and 0.75 kV/cm. However, *E*_*mid*_ obtained by sigmoidal fitting (Fig. 5c) decreased by only 8–9% compared to saline control. For cell survival (Fig. 5b), 35 mM lidocaine had a much more pronounced effect, significantly reducing survival at 1 and 1.5 kV/cm compared to control. Moreover, the effect was pH-dependent: the group with pH adjusted to 7.3 showed a 40% decrease in *E*_*mid*_ for survival, while the group without pH adjustment (pH 6.9) showed a 25% decrease in *E*_*mid*_ compared to control.

### Illustrating the impact of lidocaine on reversible and irreversible electroporation volumes using a simplified numerical model

**Fig. 6:**
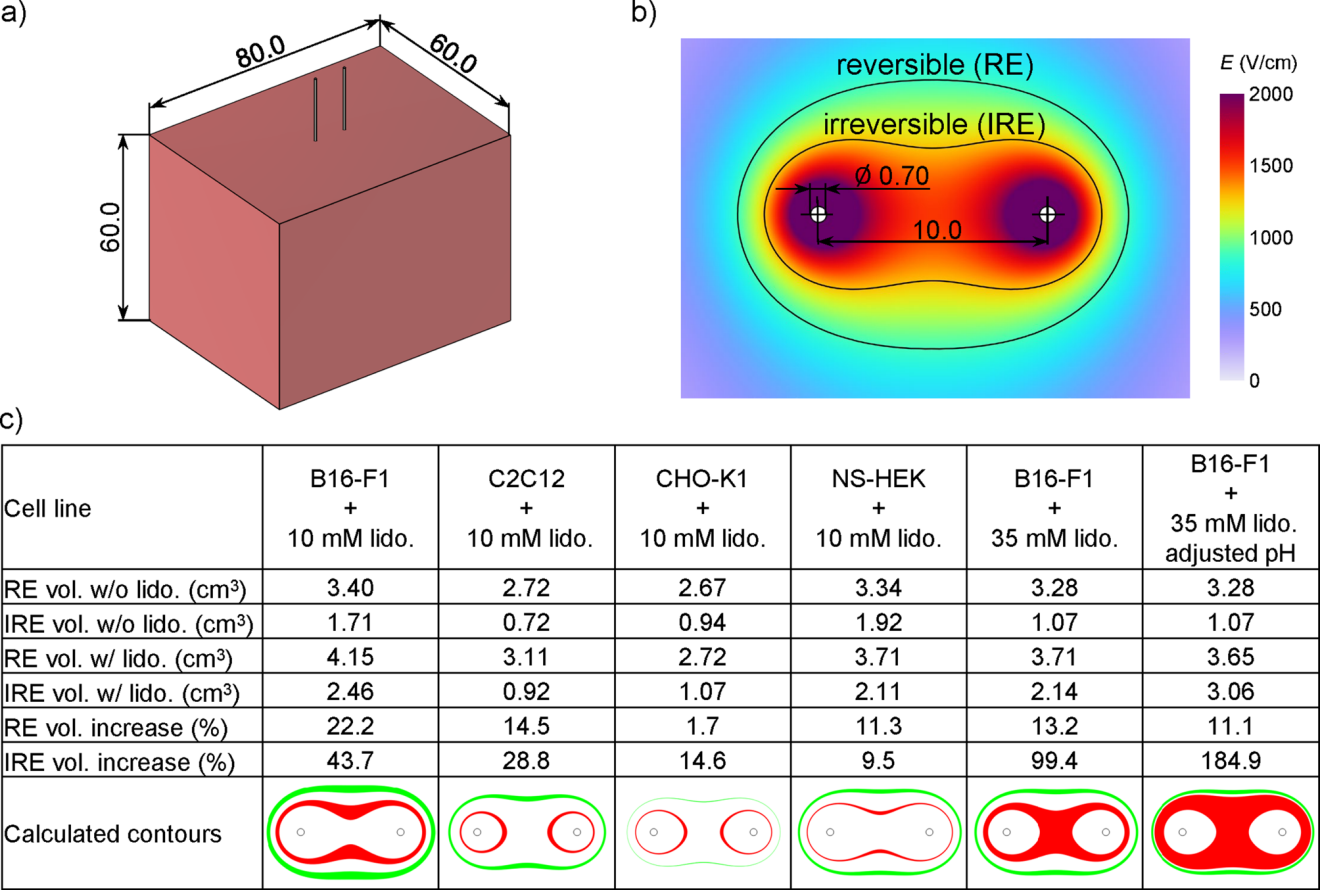
Calculations of the increase in the reversible (RE) and irreversible (IRE) electroporation tissue volumes due to lidocaine. (a) Geometry of the numerical model of tissue with two needle electrodes and indicated dimensions (in mm). The electrodes are inserted 30 mm into the tissue. (b) Cross-sectional view of an example calculation illustrating the electric field distribution and highlighting the regions of RE and IRE. (c) Calculated values of the increase in RE and IRE tissue volumes due to lidocaine. Rows 1 and 2 show the absolute volumes of RE and IRE without lidocaine (saline control). Rows 3 and 4 show the absolute volumes of RE and IRE with added lidocaine. Rows 5 and 6 show the relative increase in RE and IRE volumes with the addition of lidocaine. Row 7 shows representative cross-sections, with green indicating the calculated increase in RE volume and red indicating the increase in IRE volume due to lidocaine.

Our experimental results indicated that lidocaine decreased the *E*_*mid*_ field strength for membrane permeabilization to a modest extent (by 1−16% compared to control) at both tested concentrations. In contrast, lidocaine decreased the *E*_*mid*_ for cell survival in a markedly concentration-dependent manner: 10 mM lidocaine decreased *E*_*mid*_ by 5−18%, while 35 mM lidocaine decreased *E*_*mid*_ by 25−40%. To gain a better understanding of how this decrease in *E*_*mid*_ would translate into an increase in volume corresponding to reversible (RE) and irreversible (IRE) electroporation at a tissue level, we used a simplified numerical model (Fig. 6a). We calculated the electric field distribution around two needle electrodes (Fig. 6b) and determined the volume, where electric field was higher than *E*_*mid*_ for permeabilization (for RE) and *E*_*mid*_ for survival (for IRE). We considered only *E*_*mid*_ values obtained in experiments with Tyrode buffer. The model was designed as a simplified representation of the tissue and was not intended to capture the full complexity of the biological structure or conductivity changes due to electroporation. As such, the calculated RE and IRE volumes should be interpreted as approximate estimates that illustrate the expected trends, rather than as clinically predictive values.

The calculated values of the increase in RE and IRE tissue volumes due to lidocaine are listed in Fig. 6c. At 10 mM lidocaine concentration, the largest increase in RE volume was observed with B16-F1 cells (22.2%), followed by C2C12 cells (14.5%) and NS-HEK cells (11.3%), with CHO-K1 cells showing the smallest increase (1.7%). For IRE volumes at the same lidocaine concentration, B16-F1 cells again showed the largest increase (43.7%), while the effects were progressively smaller for C2C12 (28.8%), CHO-K1 (14.6%), and NS-HEK cells (9.5%). In experiments with B16-F1cells, increasing the lidocaine concentration to 35 mM somewhat increased the RE volume (13.2%, and 11.1% for the groups without and with adjusted pH, respectively). In contrast, this increased concentration profoundly increased the IRE volume by 99.4% and 184.9% compared to saline control, for the groups without and with adjusted pH, respectively.

## Discussion

### Revisiting the effect of lidocaine on electroporation outcome: challenging previous in vitro studies

Previous in vitro studies showed a profound effect of lidocaine on electroporation outcomes^12,13^. Specifically, Grys et al.^12^ demonstrated that 10 mM lidocaine reduced the electric field required to achieve 50% membrane permeabilization (detected by calcein uptake) and 50% cell survival (detected by fluorescein diacetate and ethidium bromide) in AT-2 rat prostate carcinoma cells by 62% and 39%, respectively. These findings indicated that lidocaine facilitates electroporation by significantly lowering the required electric field, with a more pronounced effect on membrane permeabilization than cell survival. The authors attributed this effect to the ability of lidocaine to alter the surface charge of the cell membrane, as similar reductions in electroporation thresholds were observed with cationic dyes, such as 9-aminoacridine (9-AAA) and toluidine blue. Note that different effects were observed among various local anesthetics, with procaine exhibiting a more pronounced effect than lidocaine. Similarly, Sherba et al.^13^ reported that 10 mM lidocaine significantly enhanced irreversible electroporation in NIH-3T3 mouse fibroblasts (at a specific electric field strength the percentage of cells killed increased by up to ~ 60% in lidocaine-treated group), as measured by counting the number of adherent cells. This enhancement was attributed to the lidocaine’s inhibition of ion-transporting ATPases, impairing the recovery of intracellular ionic homeostasis after electroporation. This interpretation was supported by the observation that lidocaine eliminated the protective effect of Mg^2+^ ions on cell survival, since these ions are required for Na^+^/K^+^ ATPase activation^13^.

In contrast, our findings suggest a less pronounced effect of 10 mM lidocaine on electroporation outcomes compared to previous reports. This was consistently observed across all cell lines, both in terms of membrane permeabilization (Figs. 2 and 3) and cell survival (Fig. 4), and regardless of whether cells were electroporated in low conductivity or Tyrode solution. The decrease in electric field strength required for ~ 50% cell permeabilization and ~ 50% survival (represented by *E*_*mid*_ values) did not exceed 16% and 18%, respectively, in our study (Figs. 2‒4). At specific electric field strengths, 10 mM lidocaine increased the percentage of permeabilized cells and the percentage of cells killed, but merely up to ~ 40% and ~ 30%, respectively, compared to control. Reduction in electric field threshold comparable to previous in vitro studies was observed only at the much higher concentration of 35 mM lidocaine, which resulted in 25−40% lower *E*_*mid*_ for survival in B16-F1 cells. Notably, at this higher concentration, lidocaine affected cell survival to a much greater extent than membrane permeabilization, indicating that lidocaine’s cytotoxic effect is not simply due to excessive membrane damage.

### Revisiting the effect of lidocaine on electroporation outcome: the role of experimental conditions

Our study showed a less pronounced effect of lidocaine on electroporation outcomes compared to previous in vitro studies, possibly due to more rigorous controls. In previous studies, Grys et al.^12^ used a low conductivity sucrose-based solution buffered with PBS (without reporting its pH, conductivity or osmolality). Similarly, Sherba et al.^13^ used a low conductivity sucrose-based solution buffered with HEPES (0.5 mS/cm; pH 7.4; ~300 mOsm). We showed that the addition of lidocaine to such solution considerably increases its conductivity and lowers its pH value, despite buffer presence (Fig. 2a). Since lidocaine has a pKa value of 7.6–8^34^, small changes in pH from physiological can have a considerable effect of the charge state of lidocaine^35^ and consequently its interactions with the cell membrane^16^. Furthermore, if the lidocaine stock solution is prepared without osmolality adjustments, it can result in hypotonic conditions. For these reasons, we used an isotonic lidocaine hydrochloride injection solution, added the same volume of physiological saline (0.9% NaCl) to control samples, and adjusted pH when necessary. This successfully maintained comparable pH, conductivity, and osmolality across lidocaine-treated and control groups.

While our results showed that 10 mM lidocaine’s effect on membrane permeabilization is similar regardless of the pH and conductivity variations in low conductivity solution (Fig. 2), the combined alterations in pH, conductivity, and osmolality compared to controls could have amplified lidocaine’s apparent effects in previous studies. Moreover, Grys et al.^12^ employed a microfluidic system with localized electric fields applied for 80 ms or 620 ms, significantly longer than the 100 µs pulses used in our study, resulting in greater membrane destabilization and potential temperature increases and/or electrochemical reactions. These methodological differences could explain the reduced effect of lidocaine observed in our study, highlighting the importance of consistent experimental conditions when evaluating pharmacological agents in electroporation.

In contrast to low conductivity media, adding lidocaine to Tyrode solution did not appreciably alter pH or conductivity because both the lidocaine stock and Tyrode solutions had similar conductivity, and Tyrode’s high HEPES concentration (10 mM) effectively buffered the slightly acidic lidocaine solution. Nevertheless, adding saline to control samples remained important to account for the dilution of other components within the electroporation solution upon lidocaine addition. This ensured that observed effects were attributed to lidocaine rather than changes in ionic composition. Specifically, Ca^2+^ concentration has a known impact on survival of electroporated cells^36^, with lower Ca^2+^ concentrations typically improving survival. Thus, maintaining equal dilution of Tyrode solution (containing 2 mM Ca^2+^) in both lidocaine-treated and control samples was important, as reduced Ca^2+^ concentration in lidocaine-treated samples alone could mask lidocaine’s lethal effects.

### Possible mechanisms of lidocaine’s effect on permeabilization and survival

Lidocaine may influence membrane permeabilization and cell survival following electroporation through multiple mechanisms, although it remains uncertain which are predominantly responsible for the observed effects. One proposed mechanism involves the modulation of membrane surface charge, as lidocaine is a cationic molecule that can reduce the negative surface potential, thereby enhancing membrane permeabilization. Grys et al.^12^ proposed this mechanism after observing similar effects with other cationic dyes. If surface charge was the primary mechanism, lidocaine should be considerably more effective in low conductivity solution, where fewer ions are available to screen membrane surface charge, compared to high-conductivity Tyrode solution with abundant screening ions. Our results provide mixed support for this hypothesis: we observed somewhat greater permeabilization enhancement (i.e., greater shifts in *E*_*mid*_) in low conductivity solution for CHO-K1 and NS-HEK cells, but similar effects in both solutions for B16-F1 and C2C12 cells. This suggests that while surface charge modulation may contribute to lidocaine’s effects, it is not the sole mechanism and additional cell-type-specific factors must be involved.

Experimental studies in model lipid membranes and molecular dynamics simulations further demonstrated that lidocaine interacts directly with phospholipids^16^, increasing membrane fluidity^37,38^ and bending elasticity^39^ while reducing the lipid phase transition temperature^39^. Lidocaine can also induce membrane protein clustering^37^. The nature of these interactions depends on lidocaine’s protonation state: the charged form preferentially localizes to the lipid headgroup region, while the uncharged form can penetrate into the hydrophobic lipid core and traverse the bilayer^16,40,41^. These lidocaine-membrane interactions likely make the cell membrane more susceptible to electroporation and thus contribute to increased membrane permeabilization. Additionally, since different cell types vary in their membrane lipid and protein compositions, these compositional differences could influence how lidocaine alters membrane properties and may partially explain the cell-type-specific variations in lidocaine’s effects.

Another possible mechanism involves lidocaine’s well-known interaction with voltage-gated sodium (Na_V_) channels^34^. Our study included three cell lines that express Na_V_ channels; NS-HEK are genetically engineered to express Na_V_1.5, while Na_V_ expression has also been documented in C2C12 cell line^42^ and in melanoma cells^43^, from which the B16-F1 cell line derives. Additionally, we used CHO-K1 cells, known for their low expression of voltage-gated ion channels^25^, although a subpopulation of these cells may express some Na_V_ channels^44^. In Tyrode solution, lidocaine indeed had no effect on CHO-K1 permeabilization while significantly increasing permeabilization in all three Na_V_-expressing cell lines at least at one electric field strength. However, we observed a significant increase in CHO-K1 membrane permeabilization in low conductivity solution (Fig. 2) and decreased survival in Tyrode solution (Fig. 4). This suggests that lidocaine-Na_V_ interaction may contribute to the observed effects of lidocaine, but cannot fully explain them.

Cell survival following electroporation depends on multiple biological processes, including membrane repair mechanisms, intracellular signaling, and metabolic responses^45^. Lidocaine has been reported to inhibit membrane-associated ATPases, impairing membrane recovery and restoration of intracellular ionic homeostasis after electroporation^13^. While this mechanism could contribute to the variability in lidocaine’s effects on cell survival across different cell lines, it does not align fully with our cell-type-specific findings; B16-F1 cells showed the most pronounced survival effects despite having downregulated ATPase activity^46^. Moreover, in B16-F1 cells, we observed that increasing the pH from 6.9 to 7.3 led to a pronounced reduction in the lethal electric field at 35 mM lidocaine, while membrane permeabilization was much less affected. This suggests that survival mechanisms are more sensitive to pH alterations than membrane integrity, consistent with trends observed in other cell lines^47^. This effect might also reflect the enhanced survival and function of cancer cells in a more acidic microenvironment commonly found in hypoxic tumors (pH: 6.2–6.9)^48^.

Finally, electroporation-enhanced cellular uptake of lidocaine likely potentiates its intrinsic cytotoxicity, similarly as observed with chemotherapeutic drugs in electrochemotherapy. In our previous publication we observed that lidocaine is more toxic to B16-F1 melanoma cells than to C2C12, CHO-K1 and NS-HEK^49^. This aligns with existing literature that recognizes lidocaine as a potential anticancer agent. Lidocaine’s anticancer properties have been documented across various cancer types, including lung, breast, liver, gastric, colorectal, melanoma, glioma, and tongue cancer^50^. It can act as a chemosensitizer, enhancing the efficacy of chemotherapeutic agents, including cisplatin that is commonly used in electrochemotherapy^51^. The proposed mechanisms underlying its anticancer activity are multifaceted, involving the suppression of cancer cell growth, activation of pro-apoptotic pathways, regulation of epigenetic modifications, increased generation of reactive oxygen species (ROS), modulation of key signaling pathways, inhibition of ABC transporters, and prevention of metastasis and angiogenesis. Moreover, lidocaine has been shown to regulate heat shock proteins (HSPs), matrix metalloproteinase-9 (MMP-9), GOLTI1A, p53, p38, TRPM7, and TRPV1/6, further contributing to apoptosis induction, cell cycle arrest, and ion channel regulation^50^. Notably, according to https://clinicaltrials.gov/, the anticancer potential of lidocaine is being investigated in clinical trials, including early-phase trials for pancreatic cancer (NCT04048278, recruiting) and the efficacy and prognosis of colorectal cancer (NCT04162535, unknown status)^50^.

### Clinical relevance

The practical implications of lidocaine-mediated changes in electroporation thresholds were evaluated by computing the resulting reversible (RE) and irreversible (IRE) electroporation volumes. When taking results from B16-F1 cells, 10 mM lidocaine expanded both RE and IRE volumes by 22.2% and 43.7%, respectively. At 35 mM (1%) lidocaine, the effect on RE was comparable to 10 mM lidocaine, while the effect on IRE was much more pronounced: the IRE volume nearly doubled (99.4%) in the non-pH-adjusted condition, while pH adjustment to 7.3 led to an even greater IRE expansion of 184.9% (Fig. 6c). Comparable IRE enhancement was observed by Pan et al.^14^, who used intra-arterial administration of 0.5% lidocaine before and during pulse delivery in a porcine liver model. Two protocols were tested, both applying 90 pulses of 90 µs. In Protocol 1, which employed a 2.0 cm electrode spacing, the IRE lesion volume increased by approximately 59% (from 19.9 ± 3.9 cm³ to 31.6 ± 13.0 cm³). In Protocol 2, with a wider 2.5 cm spacing, the IRE volume more than doubled (from 22.6 ± 6.4 cm³ to 46.0 ± 5.4 cm³). Importantly, administration of lidocaine during the pulse delivery ensured high local tissue concentrations during electroporation—a key difference from standard local anesthetic use where lidocaine is only administered several minutes before electroporation.

When lidocaine is used as a local anesthetic, the clinical relevance of its effects on RE and IRE volumes remains uncertain. Lidocaine tissue concentrations, several minutes after anesthetic injection, typically reach only a few mM^52,53^, well below our tested concentrations. Yet, it is important to note that these studies report total tissue concentrations of lidocaine, averaging both intracellular and extracellular compartments. Since the extracellular volume fraction is smaller, actual extracellular concentration may be higher than reported whole-tissue values, possibly approaching or even exceeding the concentrations tested in our experiments. Moreover, in fibrotic or previously irradiated tissues, where drug diffusion is often impaired, higher doses of lidocaine are injected and consequently higher local concentrations are expected^19^.

We further recognize that our experiments were conducted without chemotherapeutic agents like bleomycin and cisplatin, which are co-administered in ECT treatments. In the clinical context, even modest lidocaine-mediated increases in membrane permeability could substantially enhance the uptake and cytotoxicity of these agents, thereby improving therapeutic efficacy. Although our data showed that 10 mM lidocaine alone had only modest effects on membrane permeabilization and survival, these effects might still be clinically relevant when combined with chemotherapeutics. Furthermore, lidocaine’s anticancer and chemosensitizing properties, discussed in the preceding subsection, could act synergistically with cytotoxic drugs. Overall, the potential sensitizing effect of lidocaine in ECT warrants further investigation.

Beyond electrochemotherapy and irreversible electroporation for tumor treatment, lidocaine is clinically relevant in cardiac therapy as a class 1b antiarrhythmic agent for treating ventricular arrhythmias^17^. The FDA recently approved pulsed field ablation (PFA) for treating atrial fibrillation, and this electroporation-based technology is now being extended to ventricular applications^5^. However, plasma lidocaine concentrations in patients receiving antiarrhythmic therapy remain in the micromolar range^15^, far below our tested concentrations, making it unlikely that clinically relevant doses would significantly impact electroporation outcomes. This conclusions is supported by our findings in C2C12 cells, a model for both skeletal and cardiac muscle^54^, where 10 mM lidocaine produced only minor effects on membrane permeabilization (Fig. 3) and no significant impact on cell survival (Fig. 4).

## Conclusions

Lidocaine is widely used as a local anesthetic in clinical setting in electroporation-based medical treatments, such as electrochemotherapy and gene electrotransfer, and has been proposed as a potential electroporation sensitizer. Our study demonstrated that 10 mM lidocaine has only modest effects on electroporation outcomes, reducing electric field thresholds for reversible and irreversible electroporation by less than 16–18%, which is considerably smaller than reported in previous in vitro studies. We attribute this discrepancy to more carefully controlled experimental conditions in our study, which ensured that the pH, conductivity, osmolality, and dilution of the electroporation solution were comparable in both lidocaine-treated and control groups.

We found that the effect of lidocaine is to some extent cell type-dependent, with the most pronounced impact observed in B16-F1 melanoma cells. Variations in lidocaine’s effect on membrane permeabilization across different cell types could be influenced by membrane-level properties such as lipid composition, fluidity, and membrane protein expression profile. Further research is needed to elucidate the underlying mechanisms, for instance through molecular dynamics simulations exploring direct interactions between lidocaine and the lipid bilayer during electroporation.

Testing a higher concentration of 35 mM (1%) lidocaine in B16-F1 cells resulted in profound decrease in cell survival, reducing the threshold for irreversible electroporation by 25‒40%. Importantly, lidocaine’s impact on cell survival was disproportionately greater than its effect on membrane permeabilization, suggesting that enhanced cellular uptake across electroporated membranes potentiates lidocaine’s intrinsic cytotoxicity rather than simply causing excessive membrane damage. This mechanism aligns with lidocaine’s recognized anticancer properties in melanoma and other cancer types.

While lidocaine can considerably modulate electroporation outcomes at higher concentrations (e.g. 35 mM), the local concentration that establishes in the tissue after anesthetic injection is typically at least 10× lower, questioning the clinical relevance of this effect. Nevertheless, this effect might be important when considering combination with cytotoxic drugs, where even small enhancements in membrane permeability could considerably potentiate drug cytotoxicity. It would thus be interesting to further study potential synergistic effects between lidocaine and chemotherapeutic drugs, both in vitro and in vivo.

## Materials and methods

### Cell culture

Experiments were performed using four different cell lines: Chinese hamster ovary cells (CHO-K1, #85051005), mouse C3H muscle myoblast (C2C12, #91031101) and mouse melanoma cells (B16-F1, #92101203), all from the European Collection of Authenticated Cell Cultures. Additionally, we performed experiments on genetically engineered human embryonic kidney cells (tet-on spiking HEK, now available from ATCC, cat. no. crl-3479), which we received from the group of Adam E. Cohen, Harvard University^26,27^. Tet-on spiking HEK cells have stable expression of Na_V_1.5 channels and conditional (doxycycline-induced) expression of K_ir_2.1 channels. When grown in the presence of doxycycline, cells are able to generate action potentials and thus become spiking (S-HEK), otherwise they are nonspiking (NS-HEK).

All cell lines were grown in their corresponding growth medium with additional supplements. CHO-K1 cells were grown in Ham-F12 (#N6658). C2C12 and B16-F1 cells were grown in DMEM (#D6546 and #D5671, respectively). The growth media for CHO-K1, C2C12 and B16-F1 were supplemented with 10% fetal bovine serum (#F9665), L-glutamine (#G7513), and antibiotics Penicillin-Streptomycin (#P0781) and Gentamicin (#G1397). NS-HEK cells were grown in DMEM (#D5671) supplemented with 10% fetal bovine serum (#F2442), L-glutamine (#G7513) and antibiotics Penicillin-Streptomycin (#P0781), Puromycin Dihydrochloride (#A1113803), Blasticidin (#A1113903) and Geneticin (#10131035). The last three antibiotics were from Thermo Fischer Scientific, all other listed media were from Sigma-Aldrich.

Cells were routinely passaged every 3 to 4 days, and passages between 5 and 30 (and 3‒15 for NS-HEK cells) were used for experiments. Cells were grown in a humidified environment at 37 °C and 5% CO_2_. For experiments, cells were first trypsinized and counted. Afterwards, cells were centrifuged for 5 min/200 g and the pellet was then resuspended in the chosen electroporation solution (compositions described in section “Electroporation solutions”) to obtain a final cell density of 1 × 10^6^ cells/ml.

### Electroporation solutions

Tyrode solution was prepared in our laboratory in final composition of 125 mM NaCl (Sigma-Aldrich, #SI-71382), 2 mM KCl (Merck, #1049360550), 2 mM CaCl_2_ (Sigma-Aldrich, #SL-C4901), 1 mM MgCl_2_ (Sigma-Aldrich, #M8266), 10 mM HEPES (Merck, #1101100250), and 30 mM glucose (Merck, #MC-1083351000). Tyrode solution pH was titrated using NaOH (Merck, #1.06498.1000) or 1 M HCl (Sigma-Aldrich, #30721-M) to 7.3, which reflects the typical pH of healthy blood and extracellular fluid (commonly referred as “physiological pH”)^28^ used in in vitro experiments.

Low conductivity (LC) electroporation solution was prepared following the composition reported in a previous in vitro study^12^. LC solution consisted of 9.5% sucrose, mixed in ratio 19:1 with PBS (Gibco, #14190-094) that had been supplemented with 9 µM CaCl_2_ and 1 mM MgCl_2_.

Lidocaine HCl stock solution (20 mg/ml; 2%; 69.3 mM) was prepared by the Pharmacy of the University Medical Centre Ljubljana, Slovenia (Suppl. Fig. S1). This solution is isotonic, formulated with NaCl to adjust osmolality, and NaOH to adjust pH, and is routinely used for injections in clinical practice. The pharmacy-provided final particle concentrations are: lidocaine 0.074 mmol/ml (17.3 mg/ml), Na⁺ 0.084 mmol/ml (1.93 mg/ml), and Cl⁻ 0.156 mmol/ml (5.53 mg/ml). To achieve the desired final concentration of 10 mM (0.3%) or 35 mM (1%), 144.5–500 µl of lidocaine stock solution (or equal volume of physiological saline, 0.9% NaCl, B.Braun) was added to 855.5 µl–500 µl of the chosen electroporation solution, respectively. The pharmacological activity and stability of lidocaine in Tyrode solution was confirmed by monitoring inhibition of action potentials in S-HEK cells (Suppl. Fig. S2).

All solutions’ pH, conductivity, and osmolality values were measured using a pH meter (Metler Toledo), conductometer SevenCompact (Metler Toledo), and osmometer (Osmomat 3000, Gonotec), respectively.

### Electric pulses

Cells were exposed to 8 × 100 µs electric pulses of chosen amplitude (50–400 V), delivered by a high-frequency pulse generator L-POR (mPOR, Slovenia), through 2 mm electroporation cuvettes (VWR, #732–1136). The current and voltage were measured by the oscilloscope Wavesurfer 422, 200 MHz, the current probe CP030, and the differential probe ADP305, all from Teledyne LeCroy, USA (recording shown in Suppl. Fig. S3). The electric field to which the cells were exposed was estimated as the ratio between the applied voltage and the interelectrode distance.

### Permeabilization assay

**Fig. 7:**
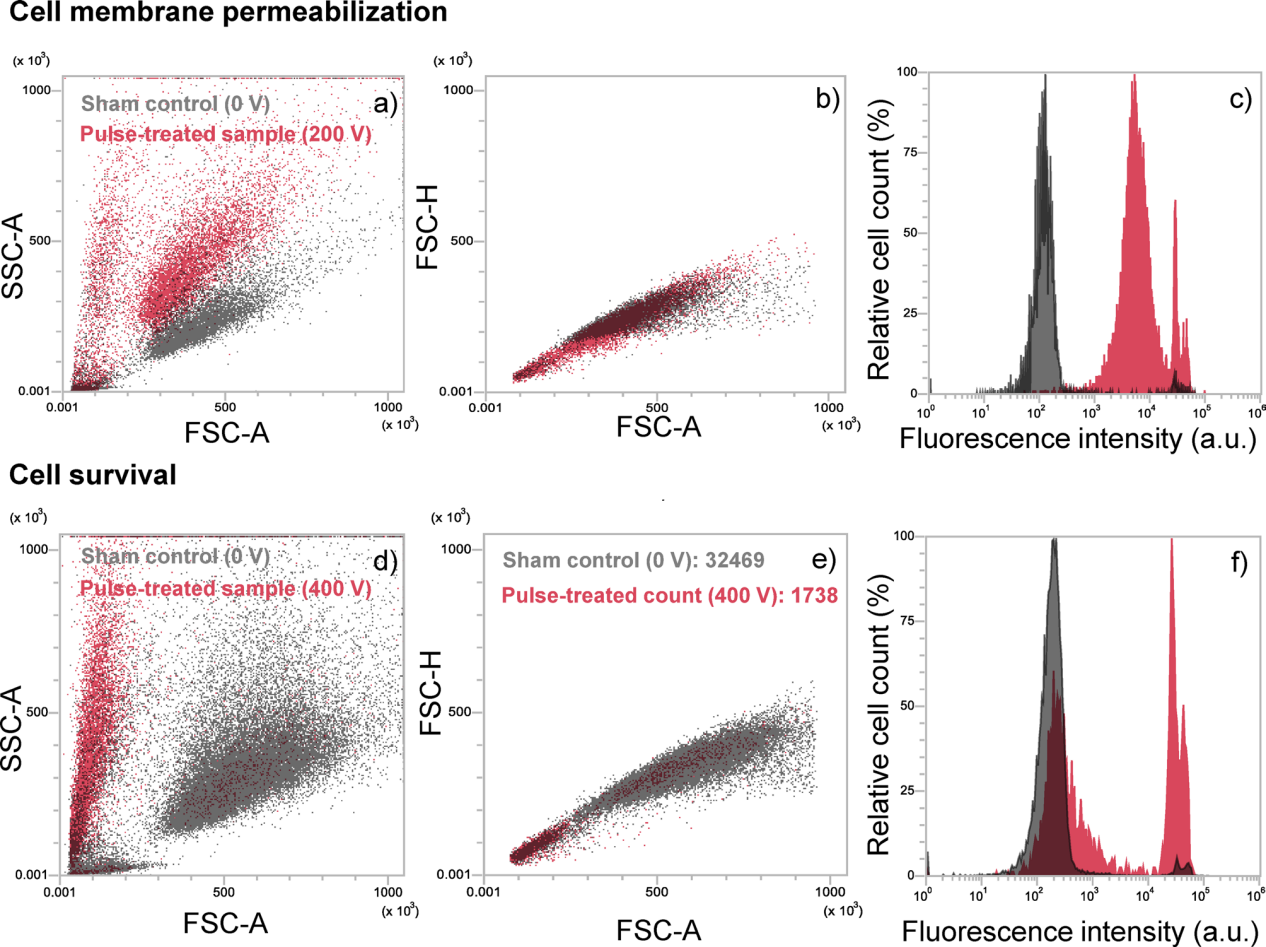
Assessment of membrane permeabilization and cell survival on a flow cytometer in the absence of lidocaine. a, d) SSC/FSC plot of untreated (sham control, 0 V) and pulse-treated cells (200–400 V). b, e) FSC area vs. height plot of cell count of untreated (sham control, 0 V) and treated cells (200–400 V). c, f) Fluorescence histogram of untreated (sham control, 0 V) and treated cells (200–400 V).

The permeabilization assay followed our earlier work^47,55–58^. Cell suspension (150 µl, 1 × 10^6^ cells/ml) in the chosen electroporation solution was mixed with propidium iodide (PI, Molecular probes, #P1304MP) in a final concentration of 100 µg/ml. 3 min after pulse application, 350 µl of electroporation solution was added to the cell suspension and the sample was transferred from the electroporation cuvette to a 1.5 ml tube. The sample was analyzed within 4 min after pulse exposure by flow cytometer (Attune NxT, Carlsbad, CA, USA) using blue laser excitation at 488 nm and detecting the emitted fluorescence through a 574/26 nm band-pass filter. 10,000 events were obtained, and data were analyzed using the Attune Nxt software. Fluorescence intensity histograms were used to determine the percentage of PI-stained cells. Gating was set according to sham control (0 V; with saline), presented in grey color in Fig. 7a–c. Measurements for each data point were repeated three times on three different days.

### Viability assay

Cell suspension (150 µl, 1 × 10^6^ cells/ml) in the chosen electroporation solution was pulsed in a cuvette. After a 10-minute waiting period, 850 µL of growth medium supplemented with 10 mM HEPES solution (Sigma-Aldrich, #SI-H0887) was added. For experiments with 10 mM lidocaine, 900 µL of suspension with 1.5 × 10^5 ^cells/mL was transferred from the cuvette to a tube, and an additional centrifugation step was applied to avoid the inherent cytotoxicity of lidocaine (for B16-F1 performed twice), observed in our previous publication^49^. Both lidocaine-treated and control samples underwent the same centrifugation procedure to ensure consistent handling across all experimental conditions. Afterwards, cells were resuspended in 850 µL of growth medium supplemented with 10 mM HEPES, and 100 µL of cells was plated in a 24-well plate (TPP Techno Plastic Products AG, Switzerland) in 1 mL growth medium. Cells were incubated at 37 °C and humidified 5% CO_2_ atmosphere. For experiments with 35 mM lidocaine, the procedure was the same, except that cells were suspended in a higher volume (5 mL) in the centrifugation step.

Propidium iodide (PI) was used to assess cell viability 24 h after pulse application. This approach was chosen over the metabolic MTS assay, used in our previous publication^49^, due to two potential limitations: (i) lidocaine may affect cellular metabolic activity and mitochondrial function, which could interfere with metabolic assay results^59^, and (ii) melanin produced by B16-F1 melanoma cells has an absorbance spectrum overlapping with MTS measurements at 490 nm, potentially overestimating cell survival^60^. Therefore, PI provided a more accurate assessment of cell survival. The protocol was similar to a previous study^60^. After 24 h, cells were harvested (attached and unattached) and centrifuged at 500 g for 5 min. The cell pellet was then resuspended in 130 µL of growth medium together with PI in a final concentration of 100 µg/ml, and cells were incubated at room temperature for 5 min. Samples were then analyzed by flow cytometer similarly as in the permeabilization assay (section “Permeabilization assay”), with one crucial difference. Instead of counting the percentage of PI-stained cells in a fixed number of detected events, we counted the number of PI-stained cells as well as the number of all cells in a fixed sample volume and determined the survival according to equation:

1 $$Cell~survival=\frac{{{N_{total}} - {N_{PI+}}}}{{{N_{total,ctrl}}}}$$

where *N*_*total*_ and *N*_*PI+*_ represent, respectively, the number of all cells and the number of PI-stained cells in an experimental group, whereas *N*_*total, ctrl*_ represents the total number of cells in sham control (0 V; with saline). This normalization was important because electroporation reduced the number of viable cells (as seen in Fig. 7d, e), so measuring PI-stained cells alone would be unreliable and would lead to considerably overestimated survival rates.

### Statistical analysis

All results are presented as mean values ± standard deviation (SD), based on at least three independent experimental repetitions performed on different days. Statistical analyses were performed using SigmaPlot 11.0 (Systat Software Inc., San Jose, CA, USA). Analysis was always carried out for each cell line separately. Data was first tested for normality using the Shapiro-Wilk test and for homogeneity of variance using Levene’s test. For datasets meeting these assumptions, One-way ANOVA was conducted, followed by Holm-Sidak’s post-hoc test for multiple comparisons (in LC solution, Fig. 2, and in experiments with 35 mM lidocaine, Fig. 5), and Holm-Sidak’s post-hoc test for comparison versus control (in Tyrode buffer, comparing 10 mM lidocaine vs. control with physiological saline, Figs. 3 and 4). If normality and/or equal variance tests failed, nonparametric ANOVA on ranks was performed, followed by Dunn’s post-hoc test. The p-value of < 0.05 was considered statistically significant.

### Numerical calculations of reversible and irreversible electroporation volumes

The distribution of the electric field within the tissue was calculated with the COMSOL Multiphysics software (version 6.3, COMSOL AB, Stockholm, Sweden) using the finite element method. A three-dimensional model was built with the dimensions shown in Fig. 6a and b. The electrodes were inserted 30 mm into the tissue. The Electric Currents physics interface was used with a Stationary study. A voltage of 2000 V was applied to the electrodes. First, the reversible (RE) and irreversible (IRE) electroporation volumes for saline control were determined. Experimentally determined electric field values corresponding to 50% permeabilization and 50% survival were used as threshold values for RE and IRE, respectively. The tissue was modelled as an isotropic and homogeneous domain, and we neglected any changes in tissue conductivity due to electroporation. Since the distribution of the electric field under such conditions is determined by Laplace’s equation for the electric potential, Δ*V* = 0, the solution is independent of specific tissue electrical properties. Thus, we used arbitrary values for tissue conductivity and permittivity, and the results can be considered representative of any isotropic homogenous tissue. The electroporated volumes with the addition of lidocaine were then calculated and the relative increase in RE and IRE volumes compared to saline control was determined.

## Electronic supplementary material

Below is the link to the electronic supplementary material.

Supplementary Material 1

## Data Availability

The data presented in this study is available on request from the corresponding author.
