# Supplementary material for: Reassessing lidocaine as an electroporation sensitizer in vitro
Source: Sci Rep. 2025 Jul 15;15:25593. doi: 10.1038/s41598-025-11695-3 (PMC12264040; doi:10.1038/s41598-025-11695-3)
Supplement: Supplementary file 1 — Supplementary Material 1 [file 41598_2025_11695_MOESM1_ESM.pdf]

Supplementary material

Reassessing lidocaine as an electroporation sensitizer *in vitro*

Anja Blažič[1], Rok Šmerc[1], Tamara Polajžer[1], Damijan Miklavčič[1], Lea Rems[1]*

[1]University of Ljubljana, Faculty of Electrical Engineering, SI-1000 Ljubljana, Slovenia
*Corresponding author (lea.rems@fe.uni-lj.si)

1. Pharmacological activity of lidocaine

In our experiments we used an isotonic lidocaine hydrochloride injection formulation, which is routinely used in clinical practice (vial shown in Fig. S1). To evaluate the pharmacological activity and stability of lidocaine in Tyrode solution, we monitored its ability to inhibit action potentials in S-HEK cells (spiking version of NS-HEK cells). We followed the experimental protocol from Batista Napotnik et. al.[1] Briefly, S-HEK cells, grown in a monolayer within Lab-Tek imaging chamber, were stained with ElectroFluor630 potentiometric dye (Potentiometric Probes, Farmington, CT, USA). A pair of parallel Pt/Ir wire electrodes, with 5 mm inter-electrode distance, was positioned at the bottom of the imaging chamber and the chamber was placed on a stage of an inverted fluorescence microscope (Leica Thunder Imager Live Cell, Leica Microsystems, Germany). Fluorescence images were captured under 635 nm LED excitation with emission detected around 700 nm (DFT51010 filter set) in time-lapse mode using LAS X software (Leica Microsystems): one image every 36 ms, 80 images, 2.8 s total duration of image acquisition. When a pulse was delivered during the time-lapse, the pulse generator was triggered by a TTL signal from the microscope at the 10th image (around 324 ms after the start of the time-lapse). Example of bright-field and fluorescence image of cells is shown in Fig. S2a.

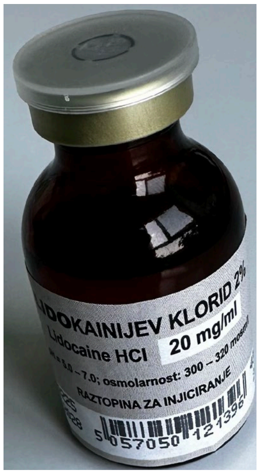

Fig. S1: Lidocaine HCl stock solution (20 mg/ml; 2%; 69.3 mM) prepared by the Pharmacy of the University Medical Centre Ljubljana, Slovenia.

The experiment proceeded as follows. First, we recorded a time-lapse without triggering the pulse, which was later used in image analysis to correct the captured signals for the dye photobleaching. Second, we recorded a time-lapse in which a single 100 µs, 150 V/cm pulse was delivered, which robustly triggered an action potential. We then added lidocaine in the desired concentration and waited for 10 minutes. Finally, we recorded the third time-lapse with pulse delivery (100 µs, 150 V/cm). Control samples were subject to the same experimental steps, just that no lidocaine was added. The captured fluorescence images were processed using a custom Matlab code to extract the relative change in fluorescence from the membranes of all cells in the field of view.[1]

Our results confirmed that 30 µM lidocaine partially inhibited $Na_V1.5$ ion channels and reduced action potential amplitude, as expected based on results from a previous study.[2] In control samples, the peak relative fluorescence change during an action potential was 0.067 ± 0.004, and this decreased to 0.043 ± 0.007 at 30 µM lidocaine (N = 3). At a higher concentration of 10 mM, lidocaine completely abolished action potential generation (Fig. S2b).

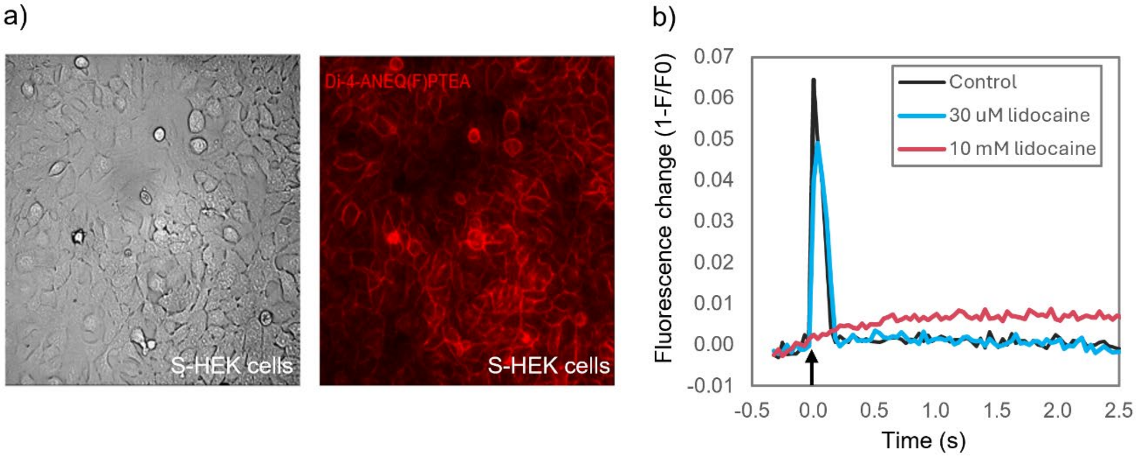

Fig. S2: Confirmation of lidocaine's pharmacological activity in Tyrode solution. a) Brightfield (left) and fluorescence image (right) of S-HEK cells stained with ElectroFluor630 potentiometric dye. b) Graph showing the time course of the relative change in the dye fluorescence indicating generation of an action potential (or absence thereof) in response to pulse application (indicated with arrow). Representative example shows how 30 µM decreased the action potential amplitude, whereas 10 mM lidocaine completely inhibited action potential generation.

2. Pulse waveform

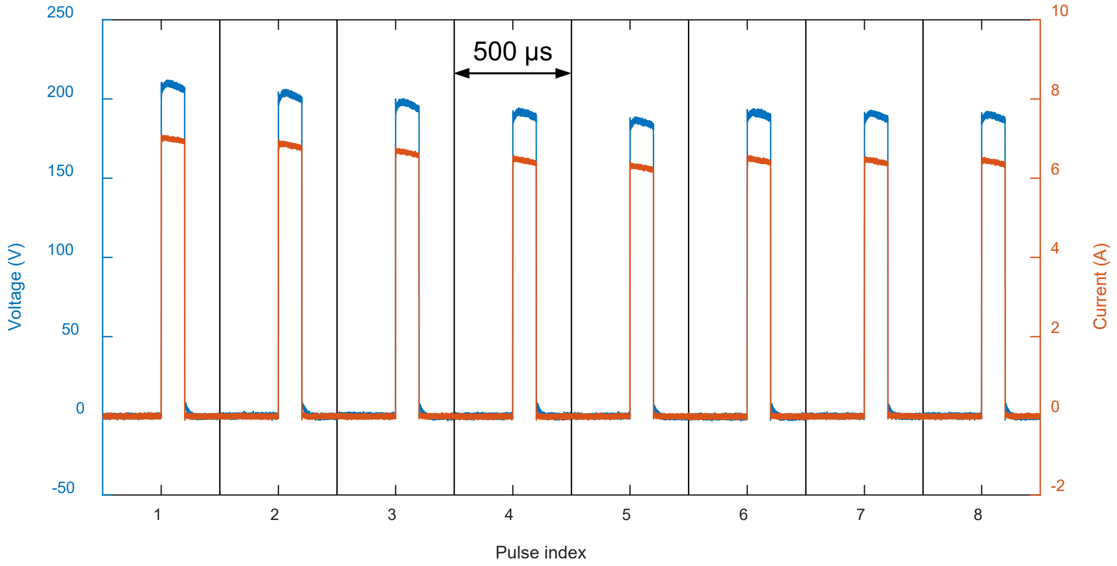

Fig. S3: Measured voltage (blue) and current (red) waveforms for eight consecutive 100 µs pulses delivered at 1 Hz, with the preset applied voltage of 200 V.

References

1. Batista Napotnik, T. *et al.* Genetically engineered HEK cells as a valuable tool for studying electroporation in excitable cells. *Sci Rep* **14**, 720 (2024).
2. Elajnaf, T., Baptista-Hon, D. T. & Hales, T. G. Potent Inactivation-Dependent Inhibition of Adult and Neonatal NaV1.5 Channels by Lidocaine and Levobupivacaine: *Anesthesia & Analgesia* **127**, 650–660 (2018).
